# Continuous and intermittent exposure of neonatal rat calvarial cells to PTHrP (1-36) inhibits bone nodule mineralization in vitro by downregulating bone sialoprotein expression via the cAMP signaling pathway

**DOI:** 10.12688/f1000research.2-77.v2

**Published:** 2013-06-13

**Authors:** Suzan A Kamel, John A Yee

**Affiliations:** 1 Department of Biomedical Sciences, School of Medicine, Creighton University, Omaha, NE, 68178, USA; 2 Department of Medical Physiology, School of Medicine, Assiut University, Asyut, 71516, Egypt

## Abstract

The development and growth of the skeleton in the absence of parathyroid-hormone-related protein (PTHrP) is abnormal.  The shortening of appendicular bones in PTHrP gene null mice is explained by an effect of PTHrP on endochondral bone growth.  Whether or not PTHrP influences intramembranous ossification is less clear.  The purpose of this study was to determine the effect of exogenous PTHrP on intramembranous ossification *in vitro*.  Neonatal rat calvarial cells maintained in primary cell culture conditions that permit spontaneous formation of woven bone nodules by intramembranous ossification were studied. The expression of PTHrP, parathyroid hormone 1 receptor (PTH1R), and alkaline phosphatase (AP) by osteogenic cells in developing nodules and the effects of PTHrP (1-36) on nodule development was determined over 3-18 days. PTHrP and PTH1R were detected colonies of osteogenic cells on culture day three, and AP was detected on day six. PTHrP and its receptor were localized in pre-osteoblasts, osteoblasts, and osteocytes, and AP activity was detected in pre-osteoblasts and osteoblasts but not osteocytes. Continuous and intermittent exposure to PTHrP (1-36) decreased the number of mineralized bone nodules and bone sialoprotein (BSP) mRNA and protein, but had no effect on the number of AP-positive osteogenic cell colonies, cell proliferation, apoptosis, or osteopontin (OPN) mRNA. These results demonstrate that osteogenic cells that participate in the formation of woven bone nodules *in vitro* exhibit PTHrP and PTH1R before they demonstrate AP activity. Exogenous PTHrP (1-36) inhibits the mineralization of woven bone deposited during bone nodule formation *in vitro,* possibly by reducing the expression of BSP.

## Introduction

Parathyroid-hormone-related protein (PTHrP) is the causative factor of humoral hypercalcemia of malignancy ^1, 2^. Although originally associated with certain types of cancer, it is now known that PTHrP is expressed by a variety of normal cell types in many fetal and adult tissues, including bone ^3, 4^. Stunted growth of the limbs and abnormalities in the craniofacial skeleton of newborn PTHrP-gene null mice served as the first evidence that PTHrP was necessary for normal skeletal development and growth ^5^. Subsequent studies using knockout and transgenic mice revealed that PTHrP influences longitudinal bone growth by controlling chondrocyte differentiation in the epiphyseal growth cartilage ^6, 7^. While development of membrane bones is also altered in PTHrP knockout mice ^5^, how the peptide contributes to intramembranous ossification has not been clarified.

The amino terminus of PTHrP has strong homology with the comparable region of parathyroid hormone (PTH) ^8^ and both peptides are physiologic ligands for the parathyroid hormone 1 receptor (PTH1R) ^9^, a member of the B family of heterotrimeric G-protein-coupled receptors. The binding of either ligand to PTH1R in bone or kidney induces a conformational change in the receptor that results in the formation of a high affinity receptor-G-protein complex that catalyzes guanine nucleotide exchange on the α-subunit of the G-protein ^10– 12^. This causes the G-protein to dissociate from the receptor-ligand complex and transduce the extracellular signal from the receptor to at least two signaling pathways, the adenylate cyclase/cAMP/protein kinase A (PKA) pathway that is mediated through the G _αs_ subunit, and the phospholipase C/diacylglycerol/protein kinase C (PKC) pathway that is mediated through the G _αq_ subunit ^13^. It is now recognized that exogenously administered amino terminal PTH has anabolic and catabolic effects in the skeleton. Whether bone formation or bone resorption occurs depends to some extent on the pattern or duration of hormone exposure. Intermittent exposure to PTH (1-34) in one or two daily subcutaneous injections increases bone formation and results in a net gain in bone mass in rats and humans ^14, 15^. By contrast, continuous infusion of PTH (1-34) preferentially stimulates bone resorption and increases serum calcium in humans ^16^ and rats ^15, 17^, with smaller effects on bone formation. The net response to continuous infusion is a decrease in bone mass ^15, 18^. Although amino terminal PTH and PTHrP bind with equal affinity to the PTH1R, intermittent PTHrP has been reported to be less effective in stimulating bone formation than equal concentrations of PTH ^15^. This finding is interesting in light of the observation that locally produced PTHrP is required for bone formation in PTH-null mice ^19^. How the contrasting effects of exogenous PTHrP are related to its physiologic function remains to be clarified.

In several tissues, including bone, it has been reported that PTHrP and PTH1R mRNAs and/or proteins are expressed by adjacent, but different, cell populations ^20– 22^. This has led to the concept that PTHrP is a paracrine factor that regulates the proliferation, differentiation, lifespan, or function of its target cells in these tissues ^23^. We previously found that PTHrP and PTH1R are co-expressed by skeletal and extraskeletal cells before and during intramembranous ossification of the chick mandible ^24^. On the basis of their temporal and spatial expression we proposed that PTHrP influences the histogenesis and/or growth of skeletal tissues in the chicken mandible via an autocrine pathway mediated by the PTH1R. The present study was designed to investigate the role of PTHrP during intramembranous ossification using neonatal rat calvarial cell cultures as an *in vitro* model. First we compared the temporal and spatial expression of PTHrP and PTH1R with the osteoblast marker protein alkaline phosphatase (AP) during the differentiation of osteoblast cell colonies and formation of woven bone nodules. Next, we examined the effect of continuous and intermittent treatment with amino terminal PTHrP on osteoblast differentiation and bone nodule formation. Finally, possible mechanisms underlying PTHrP effects on bone nodule formation were examined by assessing cell proliferation, apoptosis, and gene expression.

## Materials and methods

### Materials

PTHrP (1-36) and PTHrP (7-34) were purchased from Bachem (Torrance, CA, USA). Rabbit anti-PTHrP antibody was from Oncogene Research Products (Boston, MA, USA), rabbit anti-PTH1R antibody was from BAbCo (Richmond, CA, USA), and rabbit anti-BSP antibody was from Chemicon International (Temecula, CA, USA). Alexa fluor 488 conjugated goat anti-rabbit immunoglobulin-G (IgG) antibody was purchased from Molecular Probes, Inc. (Eugene, OR, USA). The Taqman reverse transcription kit, SYBR green polymerase chain reaction (PCR) master mix and PCR primers for glyceraldehyde 3-phosphate dehydrogenase (GAPDH), osteopontin (OP), and bone sialoprotein (BSP) were from Invitrogen (Carlsbad, CA, USA). Supersignal west pico chemiluminescent peroxidase substrate kit was from Thermo Scientific (Rockford, IL, USA). The vectastain ABC kit and diaminobenzidine (DAB) peroxidase substrate kit were from Vector Laboratories (Burlingame, CA, USA). All other chemicals were from Sigma Chemical Co. (St. Louis, MO, USA).

### Cell culture

The Creighton University Institutional Animal Care and Use Committee (Vertebrate Animal Assurance Number A3348-01) approved all animal procedures employed in this study. Pregnant Sprague-Dawley rats were purchased from Charles River, housed in the Association for Assessment and Accreditation of Laboratory Animal Care International- (AAALAC) accredited Creighton University animal resource facility, and allowed to deliver their offspring. Rat calvarial (RC) cells were isolated from calvariae of 2- to 3-day-old rat pups by digestion with crude collagenase and cultured in Dulbecco’s minimum essential medium supplemented with 10% fetal bovine serum, penicillin (100 U/ml), streptomycin (100 mg/ml), ascorbic acid (10 µg/ml), and β-glycerophosphate (5 mM). These cell culture reagents were obtained from Sigma Chemical Co. (St. Louis, MO, USA. In order to determine the effects of PTHrP on colony producing cells, cultures were initially seeded at a plating density of 75 cells/cm ^2^. Two treatment protocols were used to examine the effects of PTHrP on colony-forming RC cells. For continuous exposure, PTHrP (1-36) was present in the culture medium for the entire treatment period and, for intermittent exposure, PTHrP (1-36) was present for the first 1 or 6 hr of each 48-hr treatment period. After these brief exposures, the cells were maintained in control medium for the remainder of the 48-hr treatment cycle.

### Bone nodule assay

RC cell cultures were fixed in cold 4% paraformaldehyde, washed with distilled water, and incubated in 1% aqueous silver nitrate for 30 min in the dark. After removing the staining solution the cells were exposed to bright light for 1 hr. Mineralized bone nodules were counted at a 100X magnification using a Nikon inverted phase-contrast microscope.

### Immunohistochemistry

For confocal microscopy, RC cells were grown on glass cover slips. Specimens were collected on culture days 3, 6, 9, 12, 16, 18 and 20. Cells were fixed in cold 4% paraformaldehyde and stained for AP (see below). The cover slips were then placed in a 24-well culture plate and washed with phosphate buffered saline (PBS) containing 1% BSA and 0.1% Tween 20. Samples collected on days 16, 18, and 20 were permeabilized with 0.1% Triton X-100 in 0.1% (w/v) sodium citrate buffer. The cells were incubated with rabbit anti-PTHrP antibody (2.5 μg/ml) or rabbit anti-PTH1R antibody (1:100) overnight at 4°C, washed with the blocking buffer, and incubated with Alexa fluor 488 conjugated goat anti-rabbit antibody (1:500) for one hr at room temperature. Washed cover slips were mounted on glass slides and examined by confocal microscopy (Radiance 2000, Bio-Rad). Immunostaining was visualized using 488 nm excitation and 515/30 nm emission filters. The AP reaction product, which is autofluorescent, was visualized using 568 nm excitation and 600/40 nm emission filters. Individual images were merged using BioRad software.

### cAMP assay

Intracellular cAMP was measured using an enzyme immunoassay (EIA) kit purchased from Sigma Chemical Co. (St. Louis, MO, USA). For this experiment RC cells were plated into a 96-well plate at 10,000 cells/cm ^2^ and incubated overnight at 37°C. The experiment was initiated by adding fresh culture medium containing 0.5 mM 3-isobutyl-1-methylxanthine and 0, 0.1, 1, 10, or 100 nM PTHrP (1-36). After 10 min the medium was replaced with cell lysis reagent. cAMP was measured according to the instructions provided with the EIA kit.

### AP histochemistry

Cells that expressed AP were identified by enzyme histochemistry. Paraformaldehyde-fixed cells were incubated in 0.1 M Tris-HCl (pH 8.5) buffer containing 0.01% (w/v) naphthol AS-MX phosphate and 0.07% (w/v) fast red violet LB salt for 30 min in the dark.

### Cell proliferation assay

Cell proliferation was determined by measuring the incorporation of BrdU. RC cells plated in a 96-well plate at 200 cells/cm ^2^ were treated with 100 nM PTHrP (1-36) intermittently or continuously for four days. On day four, BrdU (10 µM) was added to each well. After 5 hr the cells were fixed with pre-cooled acidic 70% ethanol, treated with nuclease (1:100) for 30 min at 37°C, and BrdU incorporation was detected as recommended by the manufacturer of the BrdU ELISA kit.

### Apoptosis assay

Apoptotic cells were identified using an *in situ* cell death detection staining kit. Cells fixed with 4% paraformaldehyde were washed with 3% H _2_O _2_ in methanol, rinsed with PBS, permeabilized in 0.1% Triton X-100 in 0.1% sodium citrate for 2 min on ice, and incubated in TUNEL reaction mixture for 60 min at 37°C in a humidified atmosphere in the dark. After washing, the cells were incubated with peroxidase-conjugated anti-fluorescein antibody for 30 min at 37°C. Peroxidase histochemistry using DAB was used to visualize apoptotic cells. Apoptosis is expressed as the percentage of TUNEL-positive cells in RC cell colonies.

### Real-time (RT)-PCR

Total RNA was extracted from RC cells using a high purity RNA isolation kit and cDNA was synthesized using a Taqman reverse transcription kit. Quantitative PCR was performed using an ABI prism 7700 sequence detector. Amplified products were detected using SYBR Green PCR Master Mix. The forward and reverse primer sequences for OP were 5 AAAGTCGCTGACTTTGGCAG 3 and 5 AAGTGGCTACAGCATCTGAGTGT 3, and for BSP were 5 CCGGCCACGCTACTTTCTT 3 and 5 CCTGGACTGGAAACCGTTTC 3.

### Western blotting

RC cells were homogenized in lysis buffer (10 mM Tris pH 7.4, 1% SDS, 1 mM sodium orthovanadate), boiled for 5 min, and centrifuged at 15,000Xg. The protein concentration in the supernatant was measured using the bicinchoninic acid (BCA) assay. Samples containing 30 μg protein were loaded on 10% polyacrylamide minigels and electrophoresed at a constant current of 25 ma. Proteins were then transferred to nitrocellulose membrane. The blots were blocked in 5% nonfat dry milk and 0.1% Tween 20 in PBS for one hr and then incubated with a rabbit anti-BSP antibody (1:2500) overnight at 4°C. After washing, the membrane was incubated in horseradish peroxidase-conjugated donkey anti-rabbit antibody (1:40,000) [Amersham Biosciences, Amersham, UK] for one hr at room temperature. Peroxidase was detected using the supersignal west pico chemiluminescent kit and recorded on BioMax X-ray film.

### Statistics

Differences between the control and treated groups were determined by one-way ANOVA and the Bonferroni *post-hoc* test using Prism software (v4.00 for windows, GraphPad Software, San Diego, CA). A P<0.05 was considered significant.

## Results

### Expression of PTHrP, PTH1R, and AP in RC cell colonies

**Figure 1.**
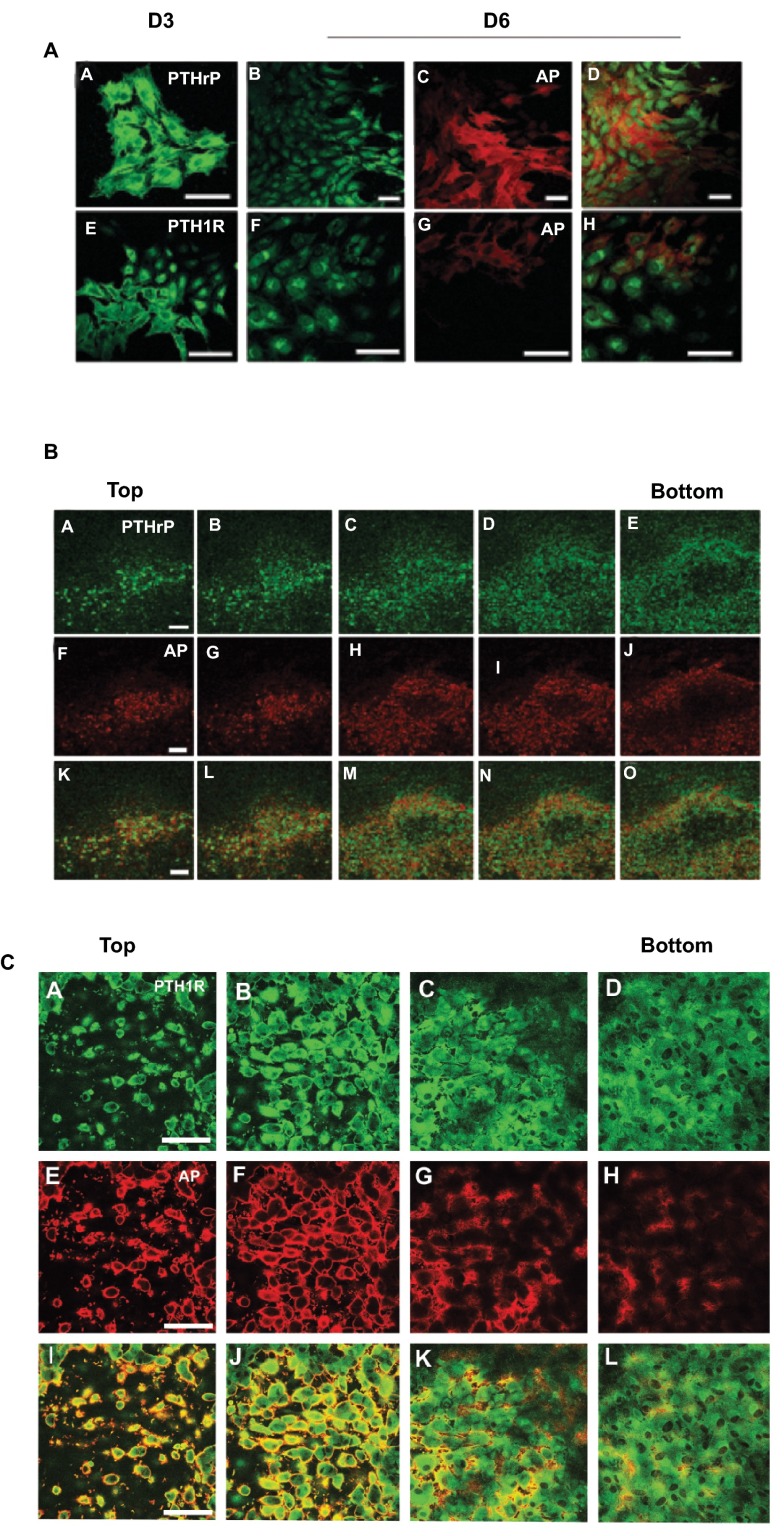
Expression of PTHrP, PTH1R, and AP in RC Cell Colonies In Low Plating Density Cultures. **A**. Cells were double-stained for PTHrP ( **A**– **D**) or PTH1R ( **E**– **H**) immunoactivity and AP after 3 and 6 days in culture. At three days cells stained positively for PTHrP ( **A**) and PTH1R ( **E**), but were negative for AP (not shown). Cells exhibiting AP activity were detected on day 6 ( **C** and **G**). The merged images of **B**– **C** and **F**– **G** are shown in **D** and **H** respectively. **B**. A single bone nodule from an 18 d culture double stained for PTHrP ( **A**– **E**) and AP ( **F**– **J**) and optically sectioned from top to bottom. The darkened area in the center of the nodule (panels C-E/H-J/M-O) indicates the mineralized region of the nodule. Cells peripheral to the mineralized region were PTHrP and AP positive. PTHrP immunostaining was less intense but persisted in cells within the mineralized area while AP staining was absent. **C**. A single bone nodule from an 18 d culture double stained for PTH1R ( **A**– **D**) and AP ( **E**– **H**) and optically sectioned from top to bottom. The darkened area in the center of the nodule (panels C,D/G,H/K,L) indicates the mineralized region of the nodule. Cells peripheral to the mineralized region were PTH1R and AP positive. PTH1R immunostaining was less intense but persisted in cells within the mineralized area while AP staining was absent.

The temporal expression of PTHrP, PTH1R, and AP in cell colonies that developed in cultures plated at low density is shown in Figure 1A. Cell colonies immunopositive for PTHrP and PTH1R were observed on culture day three ( Figure 1A, Images A and E); however, AP-positive cell colonies were not present at this time. By day six and later, some, but not all cells in the PTHrP- and PTH1R-positive colonies exhibited AP activity ( Figure 1A, Images C-D and Images G-H). A single bone nodule from a 20-day culture that was double-stained for PTHrP (Images A-E) and AP (Images F-J) and optically sectioned by confocal microscopy is shown in Figure 1B. All of the cells near the top of the nodule were positively stained for PTHrP and AP (Images A, B, F, G, K and L). Cells located deeper within the nodule in the area that contained mineralized matrix (Images C-E, H-J, and M-O) were positive for PTHrP, but AP negative. A similar pattern of staining was observed in nodules double-stained for PTH1R and AP ( Figure 1C).

### Effect of PTHrP (1-36) on bone nodule number in high initial plating density of RC cell culture

**Figure 2.**
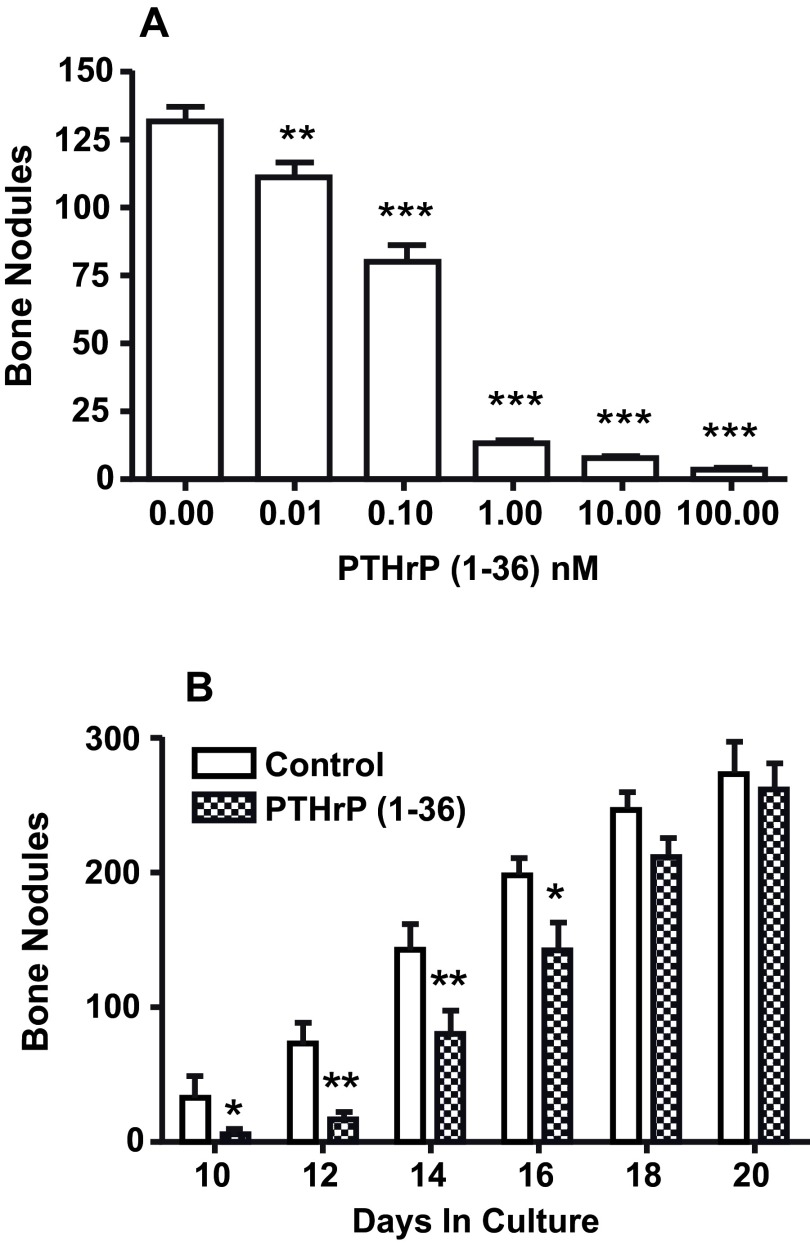
Effect of Continuous Exposure to PTHrP (1-36) and its Removal After 10 Days On Bone Nodules In High Plating Density Cultures. **A**. The effect of exposure to 0 to 100 nM PTHrP (1-36) for 18 d on the number of bone nodules in RC cell cultures. **B**. RC cells were grown in the absence (control) or presence of 1 nM PTHrP (1-36). After 10 d all cultures were maintained in control medium and the nodule number was determined on day 10, 12, 14, 16, 18 and 20. Results are the mean±SEM (n=4), *P≤0.05, **P≤0.01, and ***P≤0.001.

After 18 days, there were 131±5 bone nodules in high initial plating density control cultures. Adding PTHrP (1-36) at concentrations of 0.1 nM to 100 nM caused a dose-related decrease in the number of bone nodules ( Figure 2A). The inhibitory effect of PTHrP (1-36) on bone nodules was reversible. The number of bone nodules in cultures exposed to 1 nM PTHrP (1-36) for ten days was 80% fewer than in controls. When PTHrP-treated cultures were transferred to control culture medium the number of bone nodules rebounded and was not different from control 8 days after transfer ( Figure 2B) ^25^.

Figure 2 raw data: Effect of continuous exposure to PTHrP (1-36) and its removal after 10 days on bone nodules in high plating density cultures. The effect of exposure of RC cells to 0 to 100 nM PTHrP (1-36) for 18 days on the number of bone nodules.

### Effect of dBcAMP, forskolin, and PTHrP (7-34) on bone nodule formation

**Figure 3.**
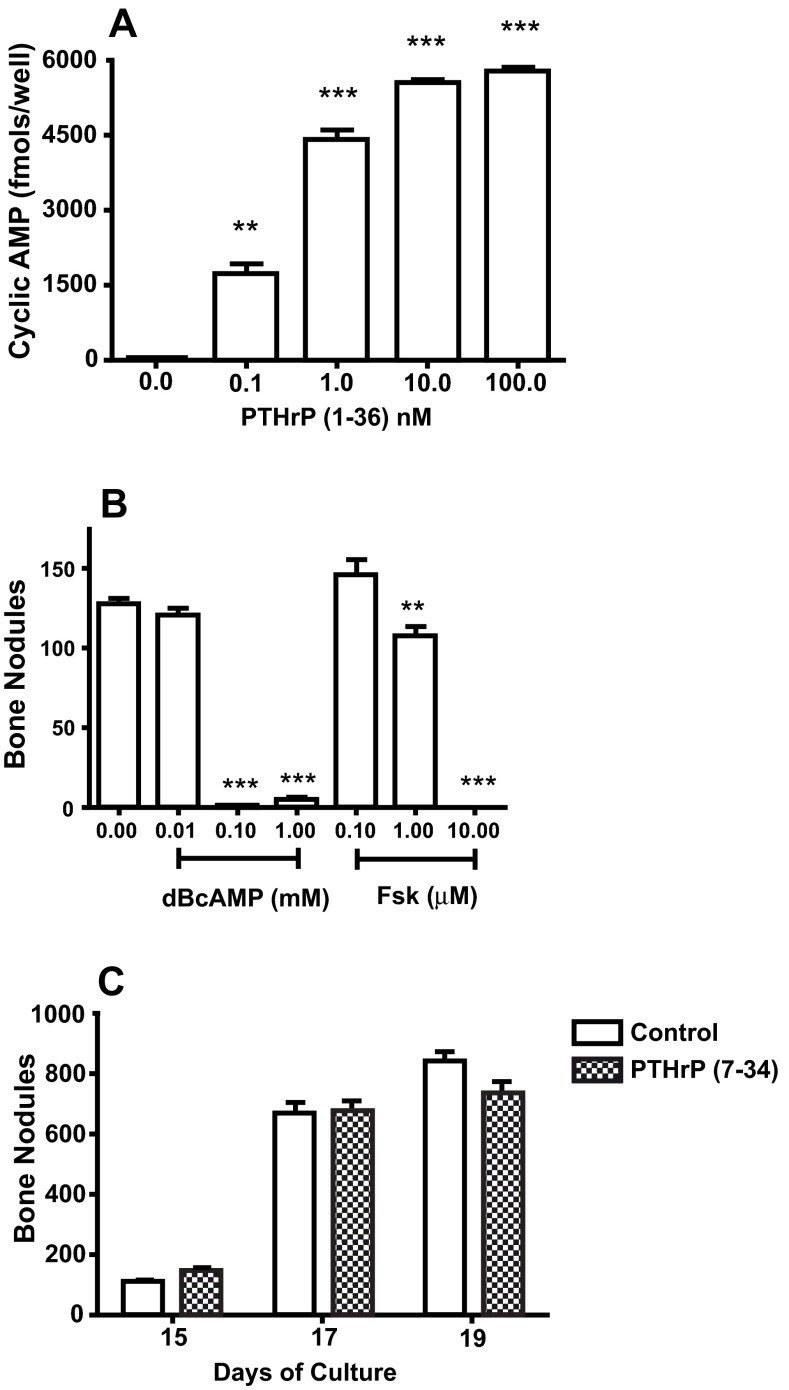
Effect of PTH1R and PKA Agonists On High Plating Density Cultures. **A**. RC cells were plated into a 96-well plate, maintained overnight, and exposed to 0 to 100 nM PTHrP (1-36) for 10 min. The amount of cAMP in each well was measured by EIA. **B**. The effect of treatment with 0 to 1 mM DBcAMP and 0 to 10 mM forskolin (Fsk) on the number of bone nodules in 18 d RC cell cultures. **C**. The effect of treatment with 50 nM PTHrP (7-34) for 15 to 19 days on the number of bone nodules in RC cell cultures. Results are the mean±SEM (n=4), **P≤0.01, and ***P≤0.001.

The concentrations of PTHrP (1-36) that decreased bone nodule formation increased cAMP ( Figure 3A). Treatment with dBcAMP or forskolin, agents that increase intracellular cAMP by diffusion through the plasma membrane or by activation of adenylate cyclase respectively, reduced bone nodule number ( Figure 3B). Treatment with 50 nM PTHrP (7-34), a PTH1R ligand that activates the phospholipase C/diacylglycerol/protein kinase C pathway but doesn’t increase cAMP, had no effect on bone nodule number ( Figure 3C).

Figure 3 raw data: Effect of PTH1R and PKA agonists on high plating density cultures. Fig3A. The amount of cAMP was measured by EIA in 98 wells plated with RC cells and exposed to 0 to 100 nM PTHrP (1-36) for 10 min. Fig3B. The number of bone nodules in 8 wells following treatment of RC cell cultures with 0 to 1 mM DBcAMP and 0 to 10 µM forskolin (Fsk). Fig3C. The number of bone nodules in RC cell cultures. RC cells were grown in the absence (control) or presence of 50 nM PTHrP (7-34).

### Effect of PTHrP (1-36) on bone nodule number and AP-positive cell colony number in low plating density RC cell cultures

**Figure 4.**
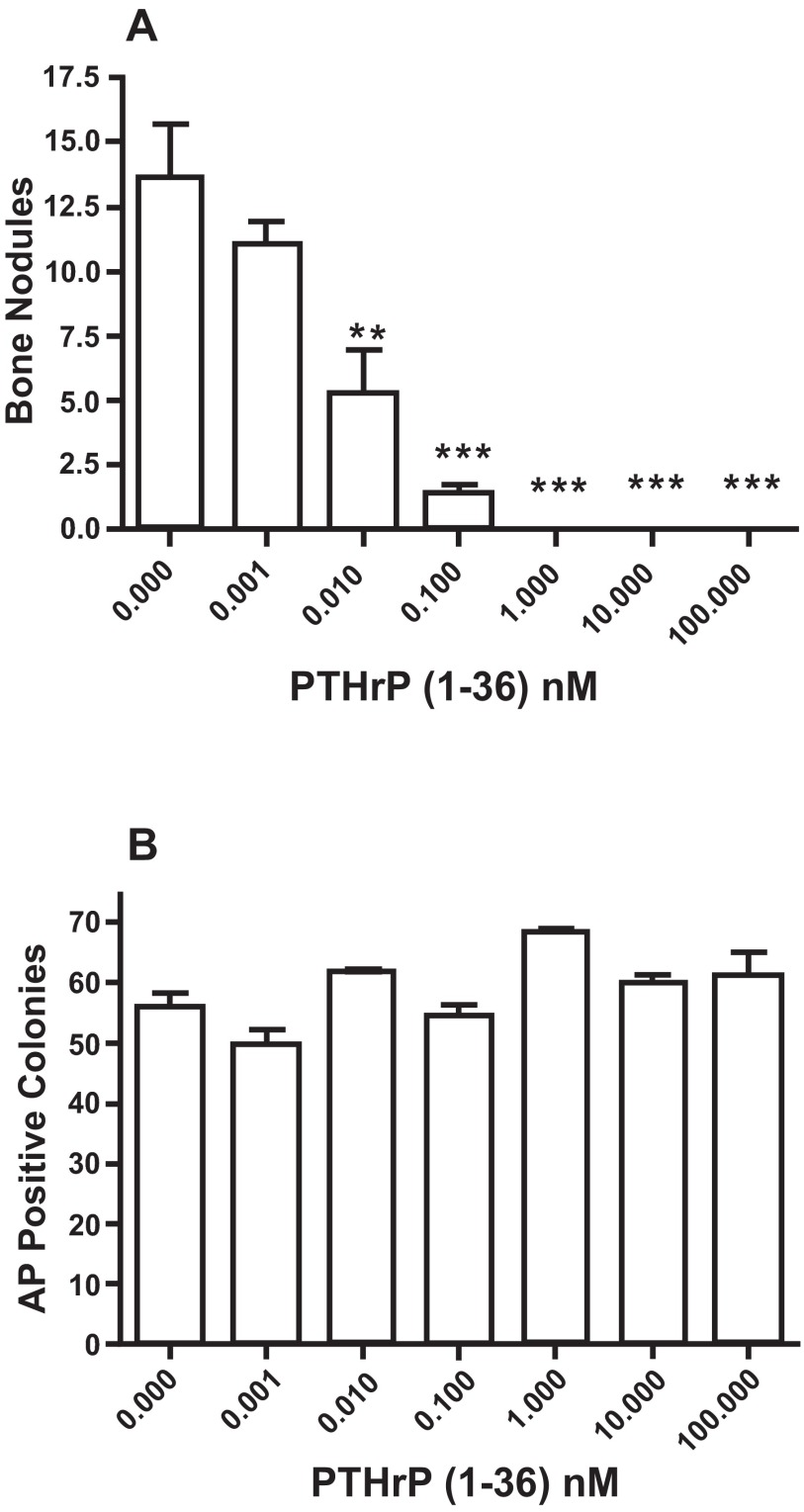
Effect of Continuous Exposure to PTHrP (1-36) On Bone Nodules and AP-Positive Osteogenic Cell Colonies In Low Plating Density Cultures. RC cells were exposed to 0 to 100 nM PTHrP (1-36) for 18 days and then fixed and stained by the von Kossa method to identify mineralized bone nodules ( **A**). or histochemically stained to identify AP-positive cell colonies ( **B**). Results are the mean±SEM (n=3), **P≤0.01, and ***P≤0.001.

**Figure 5.**
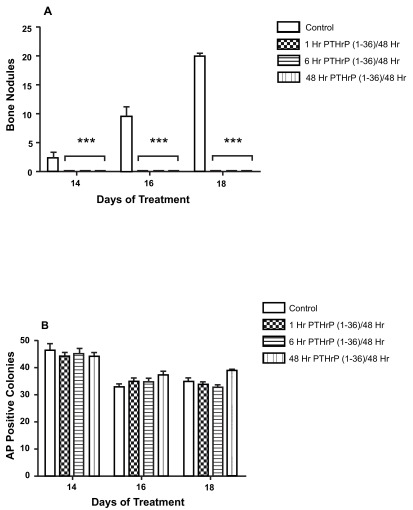
Effect of Continuous and Intermittent Exposure to PTHrP (1-36) On Bone Nodules and AP-Positive Cell Colonies In Low Plating Density Cultures. The number of bone nodules ( **A**) and AP-positive cell colonies ( **B**) in cultures exposed to 100 nM PTHrP (1-36) for 1, 6, or 48 hr during each 48 hr treatment period. Cells were exposed to a total of seven to nine 48 hr treatment cycles over 14 to18 d. Results are the mean±SEM (n=6), ***P≤0.001.

**Figure 6.**
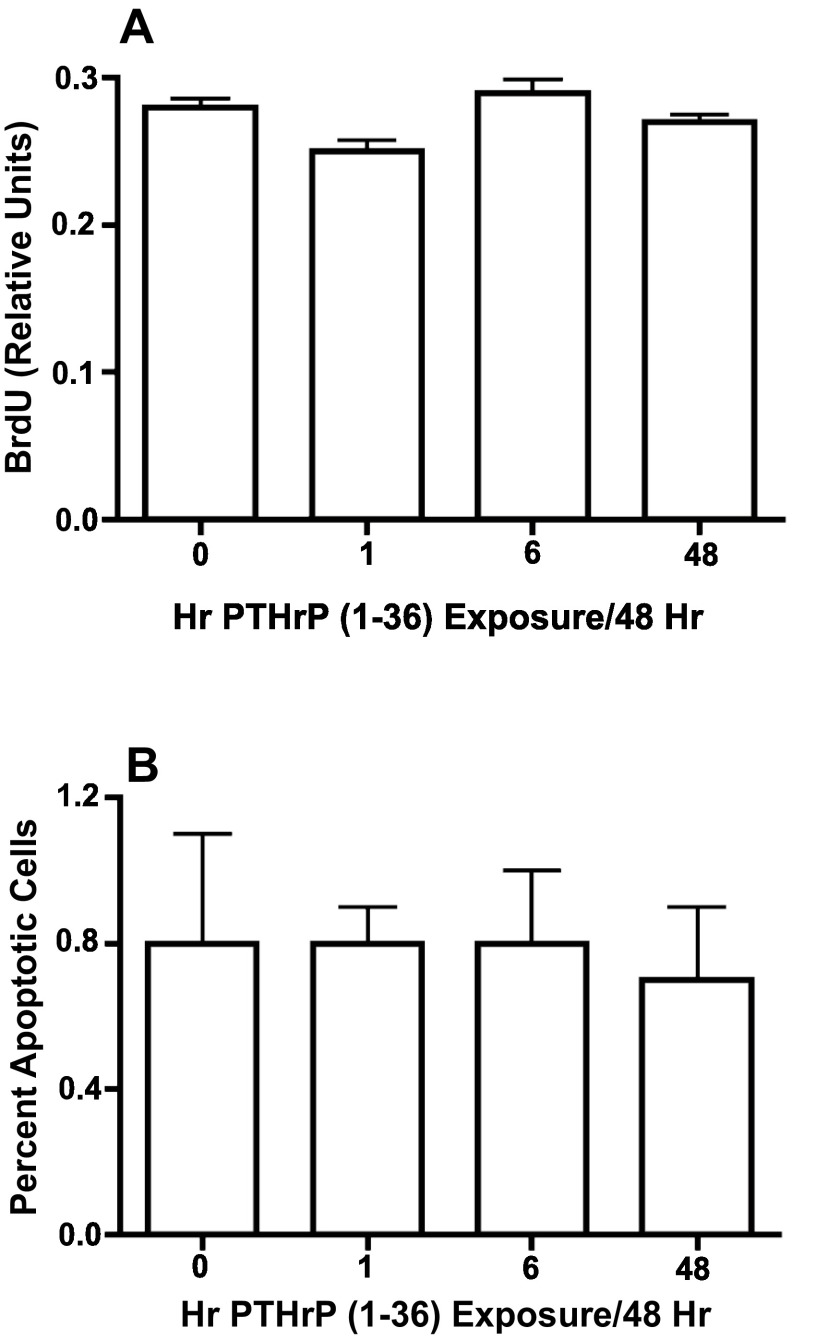
Effect of Intermittent and Continuous Exposure to PTHrP (1-36) On Cell Proliferation and Apoptosis In RC Cell Cultures. Cell proliferation was determined in RC cells treated with 100 nM PTHrP (1-36) for 1 hr, 6 hrs, or 48 hrs for two 48 hr treatment cycles and pulse-labeled with BrdU for the last 2 hrs of the experiment ( **A**). Apoptosis was measured by TUNEL staining in low plating density cultures exposed to 100 nM PTHrP (1-36) for four 48 hr intermittent treatment cycles ( **B**). Results are the mean±SEM (n=12 for cell proliferation assay and n=3 for apoptosis assay).

PTHrP (1-36) caused a dose-related decrease in the number of bone nodules in low plating density cultures ( Figure 4A), but did not affect the number of AP-positive cell colonies ( Figure 4B). To determine if the effect of PTHrP (1-36) on bone nodule development was affected by the duration of exposure to the peptide, low plating density cultures were exposed to 100 nM PTHrP (1-36) continuously or intermittently for 14, 16 or 18 days. While bone nodule formation was prevented by the three exposure regimens, none affected the number of AP-positive colonies ( Figure 5A and 5B). Continuous or intermittent exposure to 100 nM PTHrP (1-36) for four days had no effect on the proliferation of RC cells ( Figure 6A), and no effect on apoptosis was observed after exposure to PTHrP (1-36) for nine days ( Figure 6B).

Figure 4 raw data: Effect of continuous exposure to PTHrP (1-36) on bone nodules and ap-positive osteogenic cell colonies in low plating density cultures. The number of bone nodules (A) and AP positive colonies (B) in RC cells plated in low density and continuously exposed to 0 to 100 nM PTHrP (1-36) for 18 days.

Figure 5 raw data: Effect of continuous and intermittent exposure to PTHrP (1-36) on bone nodules and AP-positive cell colonies in low plating density cultures. The number of bone nodules (A) and AP-positive cell colonies (B) in RC cell cultures exposed to 100 nM PTHrP (1-36) for 1, 6, or 48 hr during each 48 hr treatment period.

Figure 6 raw data: Effect of intermittent and continuous exposure to PTHrP (1-36) on cell proliferation and apoptosis in RC cell cultures Fig6A. The number of BrdU positive RC cells to the normal number of cells following 100 nM of PTHrP (1-36) treatment for 1 hr, 6 hrs, or 48 hrs for two 48 hr treatment cycles. Fig6B. The number of apoptotic cells to the normal number of cells following 100 nM of PTHrP (1-36) treatment for 1 hr, 6 hrs, or 48 hrs for two 48 hr treatment cycles.

### The inhibitory effect of PTHrP (1-36) on bone nodule formation is immediate and reversible

**Figure 7.**
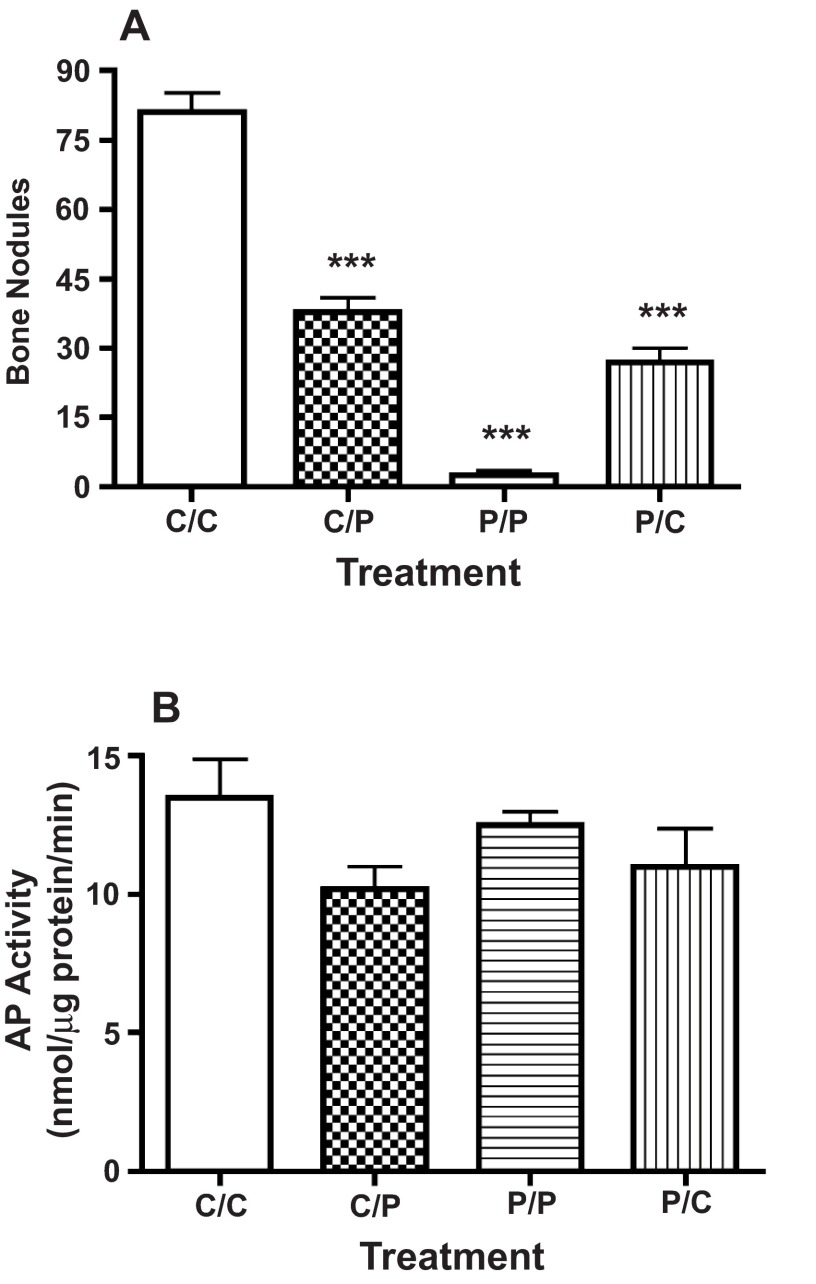
Effect of Variable Exposure to PTHrP (1-36) on Bone Nodules and AP Activity In High Plating Density Cultures. RC cell cultures were maintained in control medium for 21 days (C/C), maintained in control medium and changed to medium containing PTHrP (1-36) on day 11 (C/P), treated with 100 nM PTHrP (1-36) for 21 days (P/P), or exposed to 100 nM PTHrP (1-36) for 10 days and then transferred to control medium on day 11 (P/C). The number of bone nodules or AP activity was determined after 21 d. Results are the mean±SEM (n=4), ***P≤0.001.

The number of bone nodules that developed in high plating density cultures was reduced by approximately 50% when transferred to medium containing 50 nM PTHrP (1-36) following ten days in control conditions ( Figure 7A). By contrast, bone nodule number was increased by approximately 9-fold when cultures were transferred to control medium after an exposure to 50 nM PTHrP (1-36) for the first ten days of culture ( Figure 7A). Neither of these exposure protocols affected the AP activity ( Figure 7B).

Figure 7 raw data: Effect of variable exposure to PTHrP (1-36) on bone nodules and AP activity in high plating density cultures. RC cell cultures were maintained in control medium for 21 days (C/C), maintained in control medium and changed to medium containing PTHrP (1-36) on day 11 (C/P), treated with 100 nM PTHrP (1-36) for 21 days (P/P), or exposed to 100 nM PTHrP (1-36) for 10 days and then transferred to control medium on day 11 (P/C). The number of bone nodules (A) and AP activity (B) were determined on day 21.

### Effect of PTHrP (1-36) on osteopontin (OPN) and bone sialoprotein (BSP) mRNA and protein level in high initial plating density

**Figure 8.**
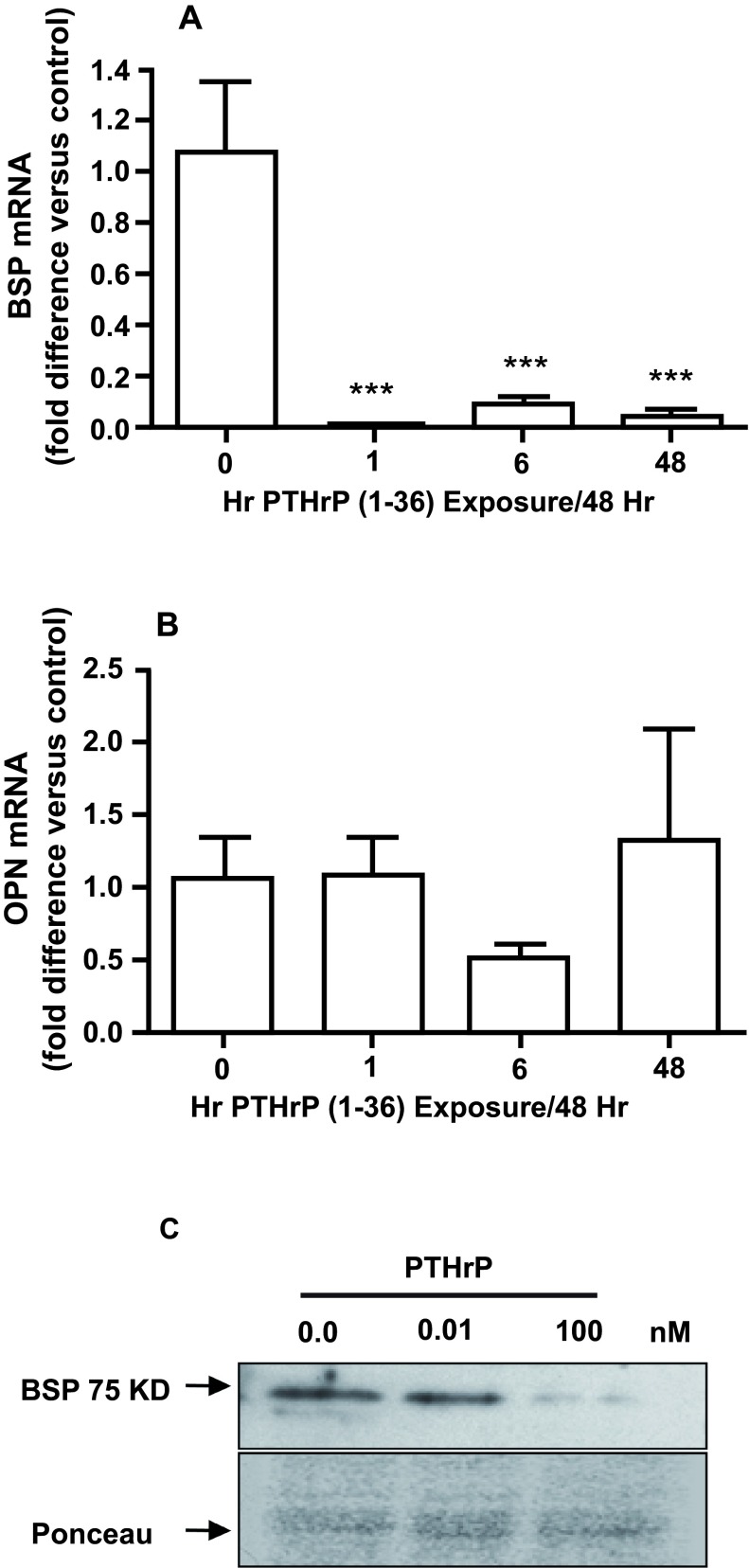
Effect of Intermittent and Continuous Exposure to PTHrP (1-36) on BSP and OPN mRNA and BSP Protein. The BSP ( **A**) and OPN ( **B**) mRNA in RC cells exposed to 100 nM PTHrP (1-36) for 1 hr, 6 hrs, or 48 hrs for seven 48 hr treatment cycles (15 days) was measured by real-time RTPCR. The effect of 0.01 and 100 nM PTHrP (1-36) on BSP in lysates of RC cells was assessed by immunoblotting ( **C**). Continuous exposure to 100 nM PTHrP (1-36) for 14 d decreased immunodetectable 75 kD BSP compared to the control and 0.01 nM PTHrP (1-36) treated RC cells. The Ponceau S-stained gel shows relative amounts of loaded protein. Real-time RTPCR results are the mean±SEM (n=3), ***P≤0.001.

Both continuous and intermittent treatment with 100 nM PTHrP (1-36) for 15 days caused a significant decrease in BSP mRNA ( Figure 8A), but did not affect OPN mRNA ( Figure 8B). Continuous exposure to 10 nM PTHrP (1-36) for 15 days also reduced the amount of BSP protein detected by immunoblotting ( Figure 8C).

Figure 8 raw data: Effect of intermittent and continuous exposure to PTHrP (1-36) on BSP and OPN mRNA and BSP protein. The BSP (A) and OPN (B) mRNA in RC cells exposed to 100 nM PTHrP (1-36) for 1 hr, 6 hrs, or 48 hrs for seven 48 hr treatment cycles (15 days) was measured by quantitative real-time RT-PCR.

## Discussion

Temporal expression of PTHrP, PTH1R, and AP associated with the maturation of cells in the osteoblast lineage during intramembranous bone formation *in vitro* was studied using primary cultures of cells isolated from calvariae of neonatal rat pups. Limiting dilution analysis has revealed that approximately 0.25–0.5% of the cells isolated from neonatal rat calvaria are osteoblast colony forming units (CFU-Os) ^26, 27^. When cultured under appropriate conditions, each CFU-O gives rise to a colony of functionally mature osteoblasts that produce a nodule of mineralized woven bone. Our rational for culturing the primary rat calvarial cells at a low plating density was to study the temporal events associated with osteoblast differentiation and woven bone formation in individual colonies in the absence of intervening cells that do not belong to a colony ^28^. Using this approach, we found small colonies of PTHrP and PTH1R immunopositive cells on the third day of culture while AP-positive cells were not detected until the sixth culture day. Although co-immunolocalization of PTHrP and PTH1R was not performed, the coincident appearance of AP in 100% of the PTHrP- and PTH1R-positive cell colonies on day six is consistent with the interpretation that the ligand and receptor are co-expressed by early progeny of neonatal rat calvarial CFU-Os. This observation confirms results reported by Kondo *et al.* ^29^ that PTHrP and PTH1R are expressed earlier than AP during osteoblast differentiation. Our observations demonstrate that osteoblasts that produce mineralized nodules of woven bone are both a source and target for PTHrP for the duration of this process. Combining the temporal/spatial expression of PTHrP and PTH1R during bone nodule formation with observations made when individual nodules were optically sectioned by confocal microscopy, our results indicate that preosteoblasts, osteoblasts, and osteocytes express PTHrP and PTH1R. This confirms our previous finding in the developing chick mandible ^24^. The co-expression of PTHrP and its receptor by osteogenic cells supports the conclusion that the peptide may serve as an autocrine/paracrine regulator of osteoblast proliferation, differentiation, survival, and/or function.

As previously reported for amino terminal PTH (1-34) ^30^, continuous exposure to PTHrP (1-36) decreases the number of bone nodules that develop in primary cultures of neonatal rat calvarial cells. Combined with the dose-related increase in cAMP stimulated by PTHrP (1-36), this establishes the functional activity of PTH1R expressed by the osteogenic cells that give rise to the woven bone nodules. As expected, several features of how PTHrP and PTH affect bone nodule formation are similar. The inhibitory effect of both peptides is dose dependent over a similar range of concentrations, the effect is reversible, and its magnitude is related to when the peptide is added to the culture. Ligand binding to PTH1R activates two intracellular signaling pathways, cAMP/PKA and PLC/DAG/PKC. That dBcAMP and forskolin, but not PTHrP 7–34, decreased bone nodule number indicates that the inhibition of nodule mineralization is mediated by cAMP. This is consistent with the finding that forskolin inhibited bone nodule formation in RC cell cultures ^31^.

The skeletal effect of exogenous amino terminal PTH and PTHrP in humans and experimental animals is affected by how the peptide is delivered. Bone resorption (catabolic effect) is the predominant effect when PTH is chronically elevated by continuous infusion ^15– 18^. By contrast, bone formation (anabolic effect) is stimulated when PTH or PTHrP are delivered in a single daily injection ^14, 15, 18^. While the anabolic effect is a consistent outcome of intermittent exposure to amino terminal PTH/PTHrP *in vivo*, this effect has been more difficult to produce *in vitro*. Ishizuya *et al.* ^32^ have reported that exposure to PTH (1-34) for the first six hours of eight consecutive 48-hr treatment cycles (17 days) increased bone nodule number and AP activity in cultures of RC cells. Bone nodule number and AP activity were decreased in cultures exposed to PTH (1-34) continuously or for one hour of each 48-hr treatment period. Using a similar design, we found that continuous as well as one- or six-hr periods of intermittent exposure to PTHrP (1-36) decreased the number of mineralized bone nodules. Interestingly, in the present study neither continuous nor intermittent treatment with PTHrP (1-36) for 14 to 18 days had an effect on the number of AP-positive cell colonies, which are derived from the same CFU-O’s that produce bone nodules. This indicates that the decline in bone nodules is not due to a decrease in the number of CFU-O’s or their ability to produce osteoblast cell colonies. Thus, the inhibitory effect of PTHrP on bone nodule formation appears to occur downstream of both cell colony formation and when AP is expressed by differentiating osteoblasts.

Datta *et al.* ^33^ reported that treatment with 1–100 nM PTHrP (1-34) stimulated cell proliferation in low-density cultures of a MC3T3-E1 subclone cell line ( Figure 1A,C). Interestingly, PTHrP (1-34) decreased proliferation in high-density cultures. This illustrates that culture conditions impact the nature of the proliferative effect of PTHrP on the cells used in their study. By comparison, we found that treatment with 100 nM PTHrP (1-36) for 5 days had no effect on cell proliferation (assessed by BrdU incorporation) in primary cultures of neonatal rat calvaria cells. Differences between the two studies that might have contributed to the varied outcomes include: the cells studied were from different species (rat versus mouse), and our experiments were done on primary cultures of rat calvaria cells while they used a subclone of a mouse calvaria osteoblast-like cell line. A potentially important difference is that the cells used in our experiments were plated, grown, and maintained in the presence of 10% FBS, whereas Datta *et al.* employed serum-starvation to synchronize cells in the cell cycle and conducted their experiments in presence of 0.5–2% FBS during the period of peptide exposure. These differences in culture conditions might be a significant factor since they noted that the stimulatory effect of PTHrP on cell number and doubling time in the presence of 0.5–2% serum was not observed in cultures supplemented with 10% serum. Differences in the observed effect of PTHrP and/or PTH peptides on cell proliferation ^34– 36^ and other endpoints of osteoblast differentiation and/or function ^37^ in different osteoblast-like cell models and under somewhat different experimental conditions is not uncommon. The fact that PTHrP prevented mineralization of bone nodules under conditions that did not affect cell proliferation is most significant in that it suggests that the effect on mineralization was not related to effects of the peptide on cell proliferation.

In this regard it is relevant that both continuous and intermittent treatment with PTHrP (1-36) decreased BSP mRNA and protein levels in RC cell cultures. BSP is a phosphoprotein that initiates nucleation of hydroxyapatite during bone matrix mineralization ^38^. The inability to mineralize woven bone matrix because of a decrease in BSP would account for the decrease in mineralized nodules identified by von Kossa staining. The finding that PTHrP (1-34) down-regulates BSP expression and inhibits biomineralization in cultures of a cementoblast cell line ^39^ is consistent with this conclusion. A functional relationship between PTH1R ligands and bone matrix mineralization is further supported by the observation that PTH and PTHrP reduce expression of *PHEX*/ *Phex*, a gene associated with phosphate metabolism, by clonal osteoblast-like UMR 106 and MC3T3-E1 cells ^40, 41^. The decrease in *Phex* expression by MC3T3-E1 cells was associated with an inhibition of the initiation and progression of matrix mineralization ^40^. While the physiologic significance of this effect is currently unknown, it is interesting that misexpression of PTHrP by osteoblasts leads to reduced mineralization of membrane bone in developing chick embryos at Hamilton Hamburger stage 44 ^42^ and that mineralization of endochondral bones is increased in PTHrP gene null mice ^5^. Taken together, these findings suggest that PTHrP may regulate the mineralization of bone matrix during skeletal morphogenesis. Moreover, it is reasonable to speculate that a transient reduction in bone mineralization might be a way to increase the local concentration of calcium and phosphate needed for other metabolic or homeostatic functions.

It is unclear why the 6-hr intermittent exposure to PTHrP (1-36) and PTH (1-34), reported to be equipotent ligands for PTH1R, had different effects in this study and in the study reported by Ishizuya *et al.* ^32^. However, it has been reported that there are differences in the ability of these ligands to interact with G-protein coupled (RG) and uncoupled (R ^0^) states of the receptor ^43^. While both ligands bind to RG with equal affinity, PTH (1-34) binds to the G-protein uncoupled receptor with a greater affinity than PTHrP. Ligands that bind stably to G-protein uncoupled PTH1R [e.g. PTH (1-34)] produce a greater cAMP response for extended times after initial binding than ligands that bind with lower affinity [e.g., PTHrP (1-36)]. Thus, a ligand that binds stably to R ^0^ has the potential to produce a downstream signal for longer times after initial binding. In addition, ligands that are bound more stably to the R ^0^ may also have a greater probability for coupling to secondary G proteins and activate alternative signaling pathways. Differential responses under seemingly identical experimental conditions might result since cAMP/PKA and PLC/DAG/PKC signaling is activated by different G-coupled proteins (G _αs_ and G _αq_ respectively). The recognition that PTHrP has a role in skeletal development was the result of observations in homozygous *PTHrP* gene null mice and PTHrP overexpressing transgenic mice ^5– 7^. The overt skeletal phenotypes exhibited by these animals indicated that PTHrP is an important regulator of embryonic endochondral ossification. It was subsequently demonstrated that longitudinal bone growth driven by interstitial expansion of the epiphyseal growth plate is controlled, at least in part, by a negative feedback loop involving Indian hedgehog protein (IHH) and PTHrP ^6, 7^. In regulating the morphogenesis and growth of endochondral bones, the source and target for PTHrP are perichondrial cells and prehypertrophic chondrocytes respectively ^44^. Although somewhat more subtle, PTHrP also influences intramembranous ossification as well as bone formation associated with skeletal modeling in postnatal mice. For example, *PTHrP* gene null mice exhibit craniofacial anomalies that may occur as a consequence of altered intramembranous ossification ^5^. Moreover, the expression of PTHrP and PTH1R mRNA and/or protein by osteoblasts has been associated with the morphogenesis of membrane bone formation in chicks ^24^, rats ^21, 45^, and rabbits ^20^. PTHrP mRNA and protein have also been detected in cultures of osteoblast-like cells isolated from fetal rat calvaria ^22^. It has been reported that PTHrP and PTH1R are expressed by morphologically and presumably functionally distinct populations of cells in the osteoblast lineage ^21, 22^. While we did not quantitate the expression of *PTHrP* and *PTH1R* in bone nodule forming colonies of osteoblasts, immunohistochemical staining results suggest that both genes are expressed by preosteoblasts, mature osteoblasts, and osteocytes. Kartsogiannis *et al.* ^20^ have reported similar findings in an experimental model of intramembranous bone formation in adult New Zealand white rabbits. This result is consistent with the conclusion that PTHrP may act as an autocrine/paracrine regulator of osteoblast differentiation and/or function during intramembranous ossification.

While there is substantial evidence to conclude that PTHrP decreases extracellular matrix mineralization during morphogenesis of intramembranous and endochondral skeletal tissues in embryos/fetuses ^5, 42^, it appears to increase bone formation by stimulating the proliferation, recruitment, and survival of osteoblasts in adult animals ^19, 46, 47^. The latter is known as the anabolic action of intermittent exposure to exogenous amino terminal PTHrP and PTH in the adult skeleton. This apparent age-associated relationship between PTHrP and its skeletal effects should be considered when evaluating the role of this protein in local regulation of skeletal cells.
