# Supplementary material for: Continuous and intermittent exposure of neonatal rat calvarial cells to PTHrP (1-36) inhibits bone nodule mineralization in vitro by downregulating bone sialoprotein expression via the cAMP signaling pathway
Source: F1000Res. 2013 Jun 13;2:77. Originally published 2013 Mar 5. [Version 2] doi: 10.12688/f1000research.2-77.v2 (PMC3901507; doi:10.12688/f1000research.2-77.v2)
Supplement: Peer review history

# Peer review: Referee response for version 2

**Published:** 2013-08-21

**Authors:** Joseph Bidwell

**Affiliations:** 1 Department of Anatomy & Cell Biology, Indiana University School of Medicine, Indianapolis, IN, USA

I approve the revised version of this manuscript without reservations.

I have read this submission. I believe that I have an appropriate level of expertise to confirm that it is of an acceptable scientific standard.

# Peer review: Referee response for version 2

**Published:** 2013-06-26

**Authors:** Daniel Bikle

**Affiliations:** 1 Department of Medicine and Dermatology, University of California/ VAMC, San Francisco, USA

The authors have improved the manuscript with the changes made in the discussion.

I have read this submission. I believe that I have an appropriate level of expertise to confirm that it is of an acceptable scientific standard.

# Peer review: Referee response for version 1

**Published:** 2013-03-19

**Authors:** Joseph Bidwell

**Affiliations:** 1 Department of Anatomy & Cell Biology, Indiana University School of Medicine, Indianapolis, IN, USA

The experiments presented in this manuscript appear to have been carefully executed. The manuscript is well written. However, the findings in this study appear to contradict an earlier report in which PTHrp induces proliferation in calvarial-derived osteoblasts (see Datta et al., 2007). The present authors do not discuss this previous result in their manuscript. These conflicting observations demonstrate the problems inherent in using *in vitro *culture systems for characterizing growth factor and hormone action on osteoprogenitors.

I have read this submission. I believe that I have an appropriate level of expertise to confirm that it is of an acceptable scientific standard, however I have significant reservations, as outlined above.

# Response

**Published:** 2013-03-22

**Authors:** Suzan Kamel

**Affiliations:** Assiut University, Egypt

Thank you Dr. Bidwell for reviewing our manuscript and we appreciate your thoughtful comments. The difference in the effect of PTHrP (1-36) on cell proliferation in our study and the outcome reported by Datta *et al*. (2007) may be related to several differences between the two studies. Datta *et al*. (2007). reported that treatment with 1-100 nM PTHrP (1-34) stimulated cell proliferation in low-density cultures of a MC3T3-E1 subclone cell line (Figure 1A,C). Interestingly, PTHrP (1-34) decreased proliferation in high-density cultures (Figure 1B). This illustrates that culture conditions impact the nature of the proliferative effect of PTHrP on the cells used in their study. By comparison, we found that treatment with 100 nM PTHrP (1-36) for 5 days had no effect on cell proliferation (assessed by BrdU incorporation) in primary cultures of neonatal rat calvaria cells. Differences between the two studies that might have contributed to the varied outcomes include: the cells studied were from different species (rat versus mouse), and our experiments were done on primary cultures of rat calvaria cells while they used a subclone of a mouse calvaria osteoblast-like cell line. A potentially important difference is that the cells used in our experiments were plated, grown, and maintained in the presence of 10% FBS, whereas Datta et al. employed serum-starvation to synchronize cells in the cell cycle and conducted their experiments in presence of 0.5-2% FBS during the period of peptide exposure. These differences in culture conditions might be a significant factor since they noted that the stimulatory effect of PTHrP on cell number and doubling time in the presence of 0.5-2% serum was not observed in cultures supplemented with 10% serum (see results section of Datta *et al*., 2007). Differences in the observed effect of PTHrP and/or PTH peptides on cell proliferation (see Sabatini *et al*., 1996; Lomri *et al*., 1997; Pasquini *et al*., 2002, and others) and other endpoints of osteoblast differentiation and/or function (see Jongen *et al*., 1993 and others) in different osteoblast-like cell models and under somewhat different experimental conditions is not uncommon. The fact that PTHrP prevented mineralization of bone nodules under conditions that did not affect cell proliferation is most significant in that it suggests that the effect on mineralization was not related to effects of the peptide on cell proliferation.

# Peer review: Referee response for version 1

**Published:** 2013-03-11

**Authors:** Daniel Bikle

**Affiliations:** 1 Department of Medicine and Dermatology, University of California/ VAMC, San Francisco, USA

This is an interesting and well written manuscript demonstrating that PTHrP blocks the mineralization of bone nodules developing in cultures of rat neonatal calvarial osteoblasts. Although similar data have been observed with PTH, and indeed both PTH and PTHrP act via the same receptor, this study demonstrates that the calvarial osteoblasts produce PTHrP as well as expressing the receptor so that they are examining a paracrine situation rather than an endocrine one.  Their results were the same whether intermittent or continuous exposure to PTHrP was utilized. Surprisingly, PTHrP did not inhibit alkaline phosphatase activity, proliferation, or apoptosis indicating that the final stages of mineralization was the point at which PTHrP was working. The authors found that BSP expression, but not OPN expression, was reduced by PTHrP, and concluded somewhat prematurely that decreased BSP expression was the mechanism for the inhibition of mineralization. There are other candidates, and the authors did not demonstrate that blocking BSP production as by siRNA would reproduce their findings with PTHrP. Thus the title of the abstract and the conclusion that BSP is the mechanism represent over interpretations. The reduction in BSP is associated with but not necessarily the reason for inhibition of mineralization following PTHrP. One would have liked to see other proteins involved with mineralization examined, and each tested for its role.

I have read this submission. I believe that I have an appropriate level of expertise to confirm that it is of an acceptable scientific standard.
